# Advanced glycation end product (AGE) targeting antibody SIWA318H is efficacious in preclinical models for pancreatic cancer

**DOI:** 10.1038/s41598-023-44211-6

**Published:** 2023-10-07

**Authors:** Gabriela R. Rossi, Ashley Jensen, Serina Ng, Zhirong Yin, Aimin Li, Anjan Misra, Daniel D. Von Hoff, Lewis Gruber, Misty Gruber, Haiyong Han

**Affiliations:** 1 SIWA Therapeutics Inc., Chicago, IL 60601 USA; 2 Molecular Medicine Division, Translational Genomics Research Institute, Part of City of Hope, 445 N. Fifth St., Phoenix, AZ 85004 USA; 3 Molecular Anatomical Pathology Cores & Biobanking Shared Resources, City of Hope, Duarte, CA 91010 USA

**Keywords:** Cancer microenvironment, Cancer therapy

## Abstract

SIWA318H is a novel monoclonal antibody that selectively targets an advanced glycation end product biomarker found in damaged/dysfunctional cells exhibiting (a) aerobic glycolysis, and (b) oxidative stress. Cells with this biomarker are dysfunctional and are associated with stresses and/or damages relating to aging, cancer and other disease processes. In this study, we evaluated the biological effects and antitumor activity of SIWA318H in preclinical models for pancreatic cancer. SIWA318H binds to pancreatic cancer cells and cancer-associated fibroblasts, as well as tumor xenografts derived from pancreatic cancer patients. Furthermore, SIWA318H induced significant antibody-dependent cell-mediated cytotoxicity (ADCC) against pancreatic cancer cells. In a humanized CD34^+^ NSG mouse xenograft model for pancreatic cancer, tumors in mice treated with SIWA318H grew significantly slower compared to those in control mice (p < 0.001). After 3 weeks of treatment with SIWA318H, the tumor growth was suppressed by 68.8% and 61.5% for the high and low dose regimens, respectively, when compared to the isotype antibody control (ANOVA p < 0.002). Moreover, a significant increase in complete remission (CR) rate was observed in mice receiving the high dose (60%, p < 0.04) or low dose (77.8%, p < 0.02) of SIWA318H treatment compared with control mice (6.7%). Immunohistochemical analyses of the tumor tissues showed a significant decrease in senescent cells in the tumor microenvironment of SIWA318H treated mice compared to that of control treated mice (p < 0.05). These results provide compelling evidence that SIWA318H is a promising novel therapeutic against pancreatic cancer.

## Introduction

Cellular senescence is a stress response that imposes a growth arrest on cancer and nonmalignant cells during cancer progression and therapy. By secreting a plethora of proinflammatory growth factors collectively termed the senescence-associated secretory phenotype (“SASP”), senescent cells can promote tumorigenesis. Moreover, the SASP from senescent cells is also able to drive therapy resistance and mediate the adverse effects of cancer therapies^1^. Because cancer therapy often leads to cell senescence, it is important to carefully consider its potential detrimental effects. Senolytics, which refers to agents that can remove senescent cells, have been proposed as a promising adjuvant approach to eliminate the adverse effects of senescent cells in the treatment of cancer and other disease^2^.

Oxidative damage accumulates in senescent cells, leading to the elevated endogenous production of advanced glycation end products (AGEs). One AGE molecule, N^Ɛ^-carboxymethyl lysine (CML), is a protein modification recognized as a biomarker for cells, such as senescent cells, damaged by accumulation of oxidized proteins^3^. The relationship between CML and cellular senescence has been demonstrated in multiple experimental systems. For example, using a telomerase-immortalized mesenchymal stem cell line, induction of cellular senescence was shown to be associated not only with typical markers, such as p16^INK4a^ and SA-*β*-galactosidase but also with increased levels of CML^4^. Also, induction of cellular senescence in normal skin fibroblasts with the dicarbonyls glyoxal and methylglyoxal resulted in increased levels of CML in parallel with the senescence marker SA-*β*-galactosidase^5^. CML oxidative post-translational modification adducts have been detected on senescence-associated cell surface vimentin with antibodies raised against senescent fibroblasts^6^.

These above data suggest that CML can serve as a selective marker for senescent cells and CML targeted agents can potentially remove senescent cells in tumors and lead to the remodeling of tumor microenvironment as well as tumor growth inhibition.

Pancreatic ductal adenocarcinoma (PDAC), the most common type of pancreatic cancer, is one of the most lethal cancers due primarily to difficulties in early detection^7^ and resistance to current therapies^8^. Recent evidence suggests that the malignant behavior of PDAC is influenced by a senescent cell-associated, strongly immunosuppressive tumor microenvironment^1^. Although senescence is known to play a tumor suppressive role, recent studies have also demonstrated the pro-tumorigenic paracrine functions of senescent cells in the tumor microenvironment^9^. Myofibroblasts in the tumor microenvironment are responsible for a desmoplastic reaction surrounding tumor glands in human PDAC^10^. Pro-fibrotic senescent fibroblasts in tumor stroma thicken and stiffen the extracellular matrix (ECM) in PDAC; such desmoplastic stiffening impedes immune infiltration and drug delivery. Hence, depletion of senescent and pro-fibrotic cells in PDAC could potentially lead to the remodeling of tumor microenvironment and antitumor activity.

In this study, we evaluated the antitumor activity of SIWA318H, a humanized senescent cell specific antibody in preclinical models for PDAC. We demonstrate that the specific binding of SIWA318H to senescent cells and its single agent antitumor activity in vitro and in vivo.

## Materials and methods

### Cell culture

Pancreatic cancer cell line PSN1 was obtained from the American Type Culture Collection (ATCC) and cultured in RPMI media supplemented with fetal bovine serum (FBS, 10%), penicillin (100 units/mL), and streptomycin (100 µg/mL). CAF08 cells are immortalized human pancreatic cancer-associated fibroblasts (CAFs) purchased from Neuromics (Minneapolis, MN) and were cultured in MSCGro media supplied by Neuromics. Cells were supplied with fresh media twice a week and monitored regularly to ensure that there was no mycoplasma contamination. The cell lines were authenticated using short tandem repeat (STR) profiling at 4-month intervals during passage.

### Antibody production and characterization

SIWA318H is a humanized IgG1 monoclonal antibody that binds strongly and specifically to carboxymethyl lysine (CML), which is an advanced glycation end product (AGE) associated with aging-related and degenerative diseases. This biomarker is found in senescent cells and other dysfunctional cells^3^. SIWA318M is the mouse monoclonal antibody from which SIWA318H was derived. The two antibodies showed almost identical dose-dependent binding to CML-modified bovine serum albumin (CML-BSA) (Supplementary Fig. S1).

SIWA318H is a 150 kDa monoclonal antibody of determined sequence, consisting of pairs of heavy and light chains complexed by disulfide bonds. SIWA318H contains a CDR binding region specific for CML. The amino acid composition of the antibody has been adjusted to avoid human immunogenic sequences. SIWA318H was expressed in CHO-K cells from a defined DNA sequence and purified by protein A affinity chromatography. The specific binding of SIWA318H to CML was verified by enzyme-linked immunosorbent assay (ELISA) and surface plasmon resonance (SPR). The production and characterization of SIWA318H was done by Fusion Antibodies PLC (Belfast, United Kingdom).

Concentration dependent binding of SIWA318H to CML-BSA were performed by ELISA. In brief, 100 ng/well of CML-BSA (Carboxymethyl lysine-BSA, Circulex, Japan) and BSA (Sigma-Aldrich, St. Louis, MO) was immobilized onto 96-well Maxisorp plates in coating buffer (0.05 M NaHCO_3_ brought to pH 9.5 by the addition of 0.05 M Na_2_CO_3_) and incubated at 37 °C for 1 h. Coating was removed and the plates were patted dry. Two hundred microliters of 3% (w/v) skim milk in phosphate buffer saline (PBS, pH 7.4) was added to each well and incubated at 4 °C overnight. The plates were then washed three times with PBS containing 0.1% Tween-20 (PBS-T). Subsequently, a serial dilution of SIWA318H and a commercially available anti-Carboxymethyl Lysine antibody (Cat# ab30922, Abcam, Cambridge, United Kingdom) in incubation buffer (PBS containing 1% Tween-20) were added to the plate and incubated for 2 h at room temperature, followed by three washes with PBS-T. To detect the antibody binding, horseradish peroxidase (HRP)-conjugated goat anti-mouse IgG (Fc specific) secondary antibody (BioRad Laboratories, Hercules, CA, Cat # 0300-0108P) or HRP-conjugated goat anti-human IgG (Fc specific) secondary antibody (Cat# A0170, Sigma-Aldrich) was added to the plates in incubation buffer for 1 h at room temperature. Following three washes with PBS-T and one wash with PBS, 100 µl of Tetramethylbenzidine (TMB) substrate was added to each well and incubated at 37 °C for 10 min. HCl (1.0 M at 50 µl/well) was then added and the plates were read immediately at 450 nm on a Tecan Sunrise microplate reader (Tecan Trading AG, Switzerland). The optical density (OD) absorbance values (with the HRP-conjugated secondary antibody only control subtracted) were plotted against the corresponding antibody concentrations using the Prism software by GraphPad (Boston, MA). Each antibody concentration was assayed in triplicates.

The binding of SIWA318H to human type III Fcγ receptor (FcγRIIIa) was tested with the same ELISA protocol as described above for CML-BSA using an FcγRIIIa specific antibody (R&D Systems, Minneapolis, MN). The EC_50_ (the concentration of antibody that gives half-maximal binding) of the binding curves were calculated by non-linear regression curve fitting using the Prism software.

### Immunofluorescence staining

PSN1 cells were seeded in LabTek™ chamber slides (ThermoFisher Scientific, Waltham, MA) at 30,000 cells/well and allowed to grow overnight. To induce cell senescence, cells were incubated with 50 µM etoposide for 24 h at which point cells were washed once with phosphate buffered saline (PBS, pH 7.4) and then allowed to recover in complete growth media for 4 days. The etoposide treated or untreated cells were then washed with PBS and fixed in 200 µL ice-cold acetone for 10 min. After fixation, cells were washed with PBS 3 times and blocked with 5% BSA for 1 h. After washed with PBS once, the cells were then incubated with SIWA318M (100 µg/mL) for two hours at room temperature followed by incubation with Alexa-488 labeled goat anti-mouse secondary antibody (1 µg/mL, ThermoFisher Scientific) for one hour. The cells were again washed with PBS 3 times and the slides were then mounted with the VECTASHIELD^®^ Antifade Mounting Medium with DAPI (Vector Laboratories, Newark, CA). The cells were visualized and imaged using a Zeiss AXIO fluorescence Imager (Zeiss, Oberkochen, Germany).

### Immunohistochemical staining of tumor tissues

The immunohistochemical (IHC) staining of PSN1 xenograft tumor tissues and patient-derived xenograft (PDX) tumors with SIWA318H was carried out using the Human-on-Human HRP-Polymer kit (Cat# BRR4056KG) by BioCare Medical, LLC (Pacheco, CA, USA) by following the procedures recommended by manufacturer. The establishment of the PDX models was described previously^11^. Briefly, PDAC tissues taken directly from patients were implanted subcutaneously in athymic nude mice. Established tumors were propagated by implantation into a new set of female mice and allowed to grow to ~ 400 mm^3^ in volume, at which point the tumor tissues were harvested and used for the IHC staining. For IHC staining, the SIWA318H primary antibody was first tagged with digoxigenin by incubating with the Digoxigenin anti-Human Linker provided in the kit. Tumor tissue sections were deparaffinized in xylene and then subjected to antigen retrieval by heating in a BioCare Diva Decloaker (Model DC2002). The slides were then blocked by incubating with the Background Punisher solution from BioCare (Tris-buffered saline solution containing purified casein, pH 7.5–7.7, Cat# IP974G20). After washing with Tris-buffered saline (TBS, pH 7.5) the slides were then incubated with the digoxigenin-tagged SIWA318H antibody (125 µg/mL) for 30 min at room temperature. The binding of the antibody to the tissues was then detected by an anti-digoxigenin secondary antibody and the MACH 2 Mouse HRP-Polymer (BioCare, Cat# MHRP520). The stained slides were scanned and visualized using an Aperio Digital Pathology Slide Scanner (Leica Microsystems, Bannockburn, IL, USA).

Dual immunohistochemical staining of p16^INK4a^ and alpha smooth muscle actin (αSMA) was performed on a Ventana Discovery Ultra IHC automated stainer (Ventana Medical Systems, Roche Diagnostics, Indianapolis, USA). Briefly, tissue blocks were sectioned at 5 µm and put on positively charged glass slides. The tissue slides were deparaffinized, rehydrated and incubated with endogenous peroxidase activity inhibitor and antigen retrieval solution. Heat inactivation was performed to prevent any cross-reactivity between each antigen detection. The antibodies against p16^INK4a^ (DAB-Brown) and αSMA (Teal) were sequentially incubated with the tissue sections. Mouse anti-p16^INK4a^ monoclonal antibody (Clone #BC42) was obtained from Biocare Medical (Pacheco, CA) and used without further dilution. Rabbit anti-αSMA monoclonal antibody was obtained from Abcam and used at 1:4,000 dilution. Following each primary antibody incubation, secondary antibodies, DISCOVERY anti-Mouse HQ or DISCOVERY anti-Rabbit HQ and detection reagents, DISCOVERY anti-HQ-HRP (all from Roche Diagnostics) were incubated according to the manufacturer specified conditions. The staining was visualized with DISCOVERY ChromoMap DAB Kit and DISCOVERY Teal Kit (Roche Diagnostics), respectively. After counterstaining with haematoxylin (Roche Diagnostics) the slides were mounted with coverslips. Slide images were acquired using a NanoZoomer S360 Digital Slide Scanner (Hamamatsu Photonics, Hamamatsu City, Japan) and viewed by NDP.view image viewer software (Hamamatsu Photonics).

The quantification of the dual immunohistochemical staining images was performed using the Visiopharm Platform (Visiopharm A/S, Hørsholm, Denmark). Briefly, Regions of Interest (ROIs) were first identified on each image (slide) and the number of cells that were positive for p^16INK4a^ within the ROIs was counted. The area of the ROIs on each image was also measured. The output cell numbers and areas for corresponding ROIs were then used to calculate the relative number of positive cells (# of cells/cm^3^ area) and compared among different treatment groups. For αSMA, the area with positive staining within the ROIs was calculated and compared to the total ROI area for each section to obtain the relative αSMA^+^ area.

### Antibody-dependent cell-mediated cytotoxicity (ADCC) assay

To determine the ADCC activity of SIWA318H against pancreatic cancer cells, an ADCC reporter assay was performed using the ADCC Reporter Bioassay Kit from Promega Corporate (CAt# G7010, Madison, WI, USA) by following the manufacturer recommended procedures. This assay uses engineered Jurkat cells that stably express Fc*γ*RIIIa receptor and contain a nuclear factor of activated T-cells (NFAT) response element driving the expression of firefly luciferase as effector cells. The ADCC activity is quantified through the luciferase produced as a result of NFAT pathway activation. Briefly, PSN1 cells (treated or untreated with etoposide as described above) were plated into 96-well plates at 12,500 cells/well and allowed to grow overnight. On the second day, a serial dilution of SIWA318H antibody as well as the effector cells (75,000 cells/well) were added to the PSN1 cells and incubated for 6 h at 37 °C in a humidified CO_2_ incubator. The Bio-Glo™Luciferase Assay Reagent (provided in the Promega kit) was then added to the cells to determine the luciferase activity by measuring the luminescence signaling using an EnVision^®^2105 multimode plate reader (PerkinElmer, Waltham, MA, USA). Luminescence signal units (RLU) of the antibody treated samples subtracted by the no antibody control were reported. Each antibody concentration was assayed in triplicate.

### Animal study in humanized NSG mice

The animal study was carried out adhering to recommendations in the NIH Guide for the Care and Use of Laboratory Animals. A protocol for the study was approved by the Institutional Animal Care and Use Committee (IACUC) at the University of Arizona, where the animal studies were carried out. Approximately 4-month-old female humanized CD34^+^ NSG mice (Hu-CD34^+^ NSG) were obtained from The Jackson Laboratory. These mice had been humanized by engrafting human CD34^+^ hematopoietic stem cells and were confirmed to be engrafted with > 25% hu-CD45^+^ peripheral blood cells. PSN1 cells (3 × 10^6^/mouse) were implanted subcutaneously into the flanks of the Hu-CD34^+^ NSG mice and allowed to grow for 10 days. On Day 10, mice were randomly assigned to three treatment groups (n = 16/group): (1) IgG1 isotype control (20 mg/kg, i.p., BIW (twice a week) × 1 followed by 10 mg/kg, BIW × 2); (2) high dose SIWA318H (20 mg/kg, i.p., BIW × 1 followed by 10 mg/kg, BIW × 2); and (3) low dose SIWA318H (10 mg/kg, i.p., BIW × 1 followed by 5 mg/kg, BIW × 2). Mice were monitored twice weekly for body weight and tumor volume by measuring the greatest longitudinal diameter (length) and the greatest transverse diameter (width) of tumor using a caliper. Tumor volume was calculated by the formula: (length × width^2^)/2. Mice were humanely euthanized when they showed clinical signs of morbidity, or their tumor volume reached 2000 mm^3^. The method of euthanasia was isoflurane overdose accompanied with cervical dislocation. Tumor tissues were collected at the end of study (Day 45 after the initiation of treatment). The data from the animal studies are reported according to the "Animal Research: Reporting In Vivo Experiments" guidelines.

### Data analysis and statistical methods

Statistical significance between two groups was evaluated using the standard two-tailed *t*-test. Two-tailed ANOVA and mixed effects models were used to evaluate the statistical differences between two treatment groups over a period of time (tumor growth curves) or a range of concentrations (ADCC assay). Logrank test was used to determine the significance of difference in survival between different treatment groups for the in vivo efficacy study. A p value < 0.05 was considered to represent statistical significance.

## Results

### SIWA318H binds specifically to CML modified BSA with high affinity

**Figure 1:**
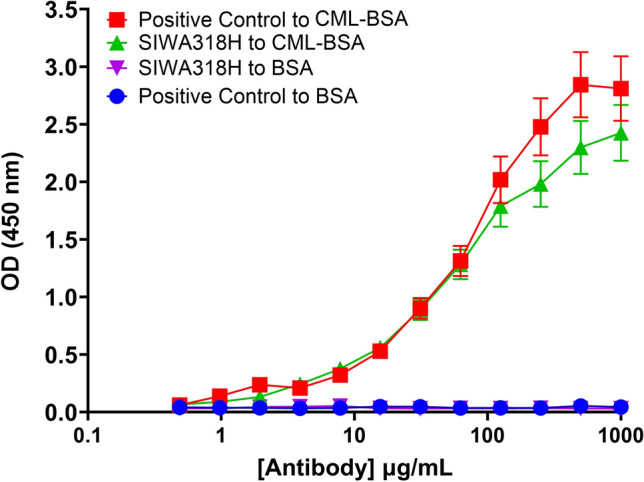
SIWA318H binds to CML-BSA in a concentration dependent manner. SIWA318H showed a concentration-dependent binding to CML-BSA that is similar to a commercially available anti-CML antibody (positive control). There was no binding to BSA detected at the highest concentration tested, indicating the high specificity of SIWA318H to the CML glycation epitope.

To determine its specificity, we tested the binding of SIWA318H to BSA and CML-modified BSA (CML-BSA) using an ELISA assay. As shown in Fig. 1, comparable binding to CML-BSA was observed with a positive control antibody (anti-Carboxymethyl Lysine antibody from Abcam) and SIWA318H while no binding was observed to BSA for either antibody demonstrating that SIWA318H is specific for CML. Similarly, SIWA318H showed robust concentration-dependent binding to CML-BSA, but not BSA, in a surface plasmon resonance binding assay (Supplementary Fig. S2).

### SIWA318 binds to senescent pancreatic cancer cells and cancer associated fibroblasts

**Figure 2:**
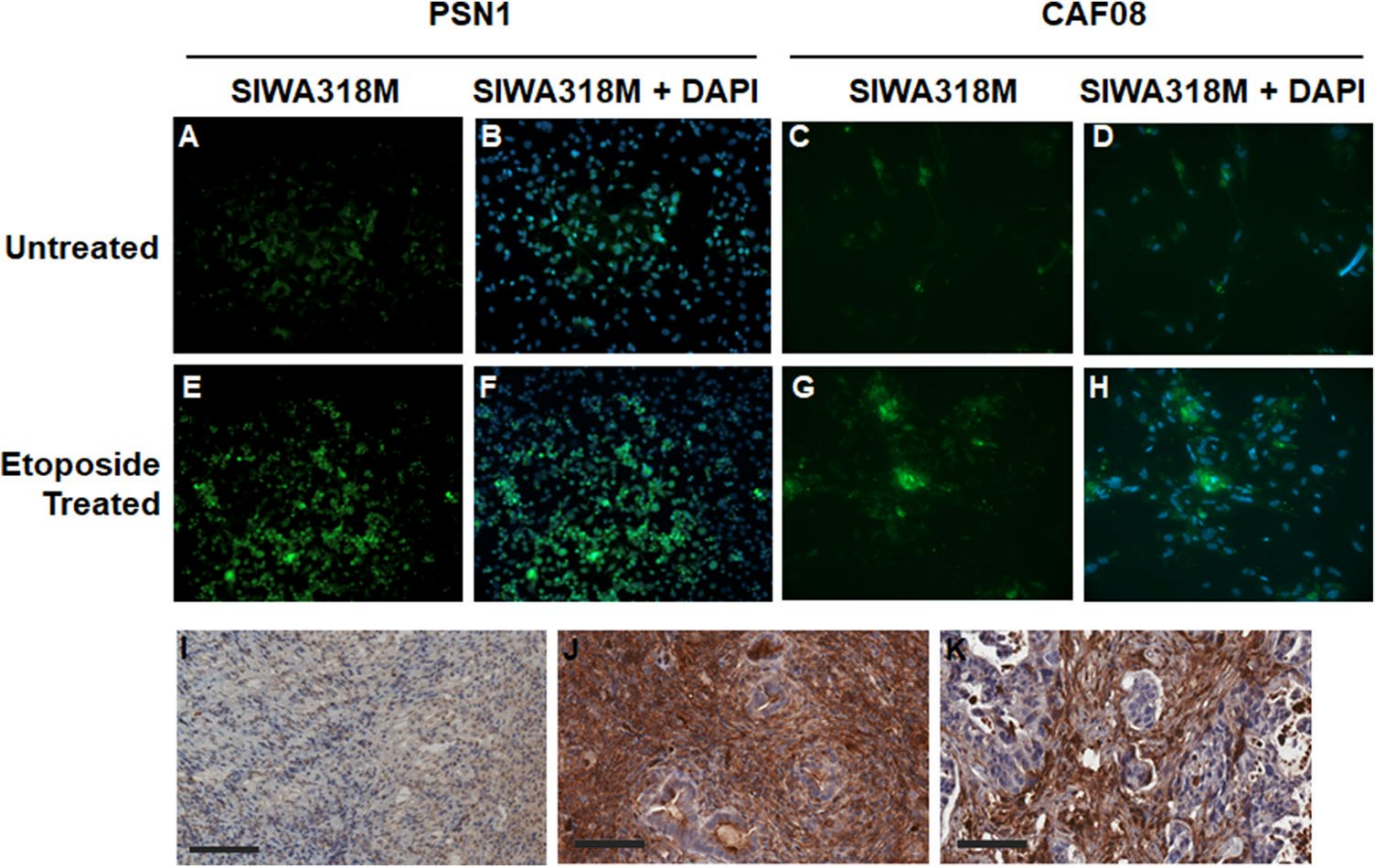
SIWA318M and SIWA318H react with pancreatic cancer cells and cancer associated fibroblasts. Immunofluorescence staining was used to detect the binding of SIWA318M to pancreatic cancer cells (PSN1) and pancreatic cancer-associated fibroblasts (CAF08) untreated (**A**–**D**) or treated (**E**–**H**) with etoposide. IHC staining was used to detect the binding of SIWA318H to the PSN1 xenograft tumor (**I**) and two pancreatic cancer patient derived xenograft tumors (**J**,**K**). Scale bar in (**I**–**K**) = 100 µm. DAPI: 4′,6-diamidino-2-phenylindole.

To investigate the reactivity of SIWA318M (the murine equivalent of SIWA318H) towards pancreatic cancer cells and stromal fibroblasts, we performed immunofluorescence staining of the antibody in PSN1 (pancreatic cancer cells) and CAF08 (pancreatic cancer-associated fibroblasts) cells with or without the treatment of etoposide, a topoisomerase inhibitor known to induce cellular senescence^12^. As shown in Fig. 2, without etoposide treatment, SIWA318M showed moderate reactivity to a small number of the cells (Fig. 2A–D). With etoposide treatment, the immunofluorescence intensity and percent of cells stained positive were increased considerably (Fig. 2E–H), which is consistent with the induction of senescence by etoposide.

To further verify the reactivity of SIWA318H towards pancreatic tumors, we performed immunochemical staining with SIWA318H in a PSN1 xenograft tumor and two pancreatic cancer PDX tumors. As can be seen in Fig. 2I, the PSN1 tumor cells showed moderate reactivity to SIWA318H, similar to PSN1 cells grown in vitro (Fig. 2A). In both PDX tumors, the stromal cells showed intense reactivity whereas the tumor cells showed relatively weak and patchy staining (Fig. 2J,K), indicating that in patient tumors the majority of the senescent cells are in the stroma compartment. SIWA318H mainly showed membranous and cytoplasmic staining with occasional nuclear staining in the PDX tumors whereas in the PSN1 tumors the staining intensity in cytoplasm/nuclei was much stronger than that on cell surface, which is again consistent with the cultured PSN1 cells in vitro. The difference in the staining pattern between PDX tumors and the PSN1 tumor is probably due to the difference in stromal content between the two tumor types (PDX tumors have a much higher stroma content than the PSN1 tumor).

### SIWA318H binds to human FcγRIIIa and induces antibody-dependent cell-mediated cytotoxicity

**Figure 3:**
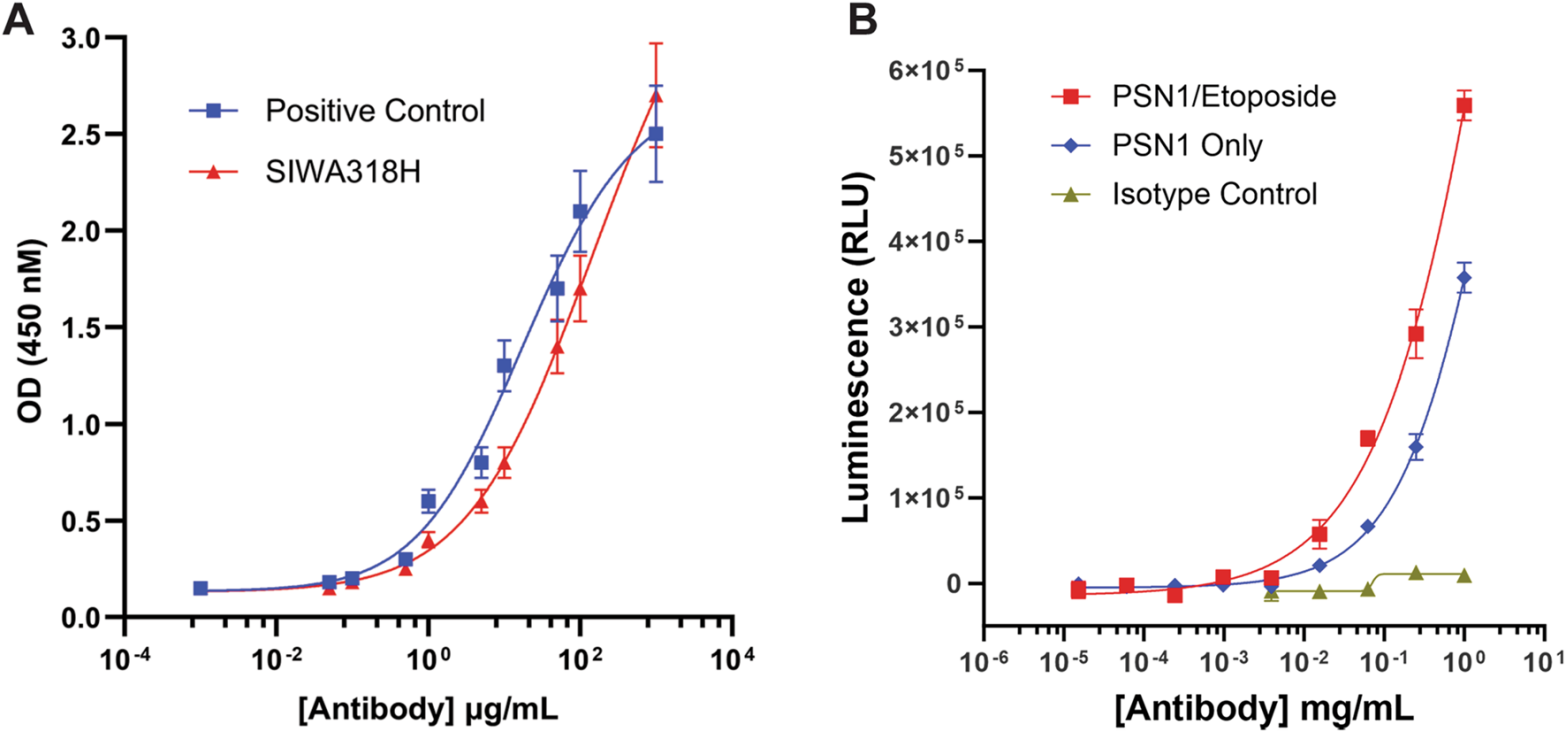
SIWA318H binds to human FcγRIIIa and induces ADCC. (**A**) Concentration-dependent binding of SIWA318H to human FcγRIIIa. A commercially available reference antibody from R&D Systems served as a positive control. (**B**) Concentration-dependent induction of ADCC activity by SIWA318H against etoposide treated and untreated PSN1 cells.

Therapeutic antibodies have been shown to interact with the immune activating Fcγ receptor, FcγRIIIa, and induce antibody-dependent cell-mediated cytotoxicity (ADCC) in solid tumors^13^. To determine the binding affinity of SIWA318H towards FcγRIIIa we performed an FcγRIIIa binding immunoassay. As shown in the Fig. 3A, SIWA318H binds to human FcγRIIIa at an affinity similar to that of the positive control antibody (anti-CML monoclonal antibody, R&D Systems) (EC_50_: 59 vs. 13 µg/mL). Consistent with its binding activity towards FcγRIIIa, SIWA318H demonstrated a concentration dependent cytotoxicity against PSN1 cells in an ADCC Reporter Bioassay (Fig. 3B). Treatment of PSN1 cells with etoposide further increased the ADCC activity, consistent with the finding that SIWA318H binds to senescent cells at a greater affinity (Fig. 2).

### SIWA318H demonstrates significant antitumor activity in a humanized mouse xenograft model

**Figure 4:**
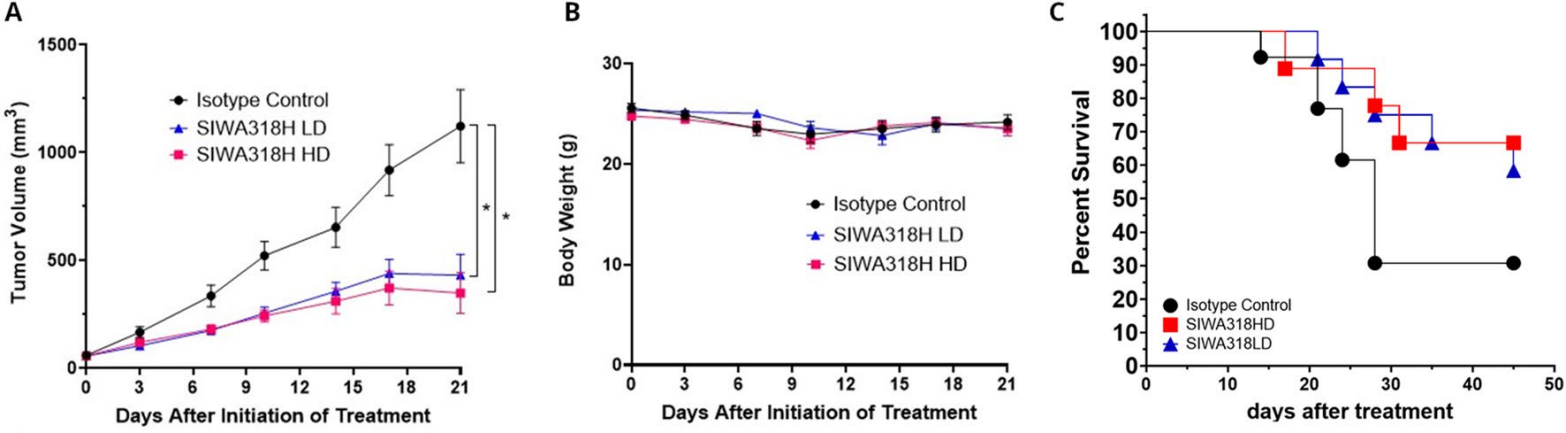
SIWA318H exhibits potent antitumor activity in the PSN1 xenograft model using humanized NSG mice. (**A**) Tumor growth was suppressed by both low dose (LD) and high dose (HD) SIWA318H treatment. *p < 0.001. (**B**) No significant difference in mouse body weight was observed between SIWA318H treated mice and the isotype control antibody treated mice. (**C**) SIWA318H treatment improved mouse survival (n = 13, 12, and 9 for the isotype, LD, and HD groups, respectively).

To further evaluate the antitumor activity of SIWA318H in vivo, we conducted an efficacy study in the PSN1 xenograft model using NSG mice humanized with CD34^+^ hematopoietic stem cells. These mice were confirmed to have robust multi-lineage engraftment of human immune cell populations (T cells, B cells, and myeloid cells) and confirmed to have at least 40% (average 68.7%) human CD45^+^ in peripheral blood. The mice were treated with either a low dose (LD) SIWA318H, a high dose (HD) SIWA318H, or an IgG1 isotype. Mice in both SIWA318H treated groups showed significant (p < 0.001) decrease in the rate of tumor growth compared to the isotope control group as shown in Fig. 4A. The reduction in tumor growth rate in the HD group appeared to be greater than that in the LD group but the difference is not statistically significant (p > 0.05). There is no significant difference in mouse body weight changes between the SIWA318H treatment groups and the isotype control group (Fig. 4B), indicating the doses and schedules of SIWA318H used in this study were well tolerated by the animals.

The tumor growth was followed up for up to 45 days after the initiation of treatment. On Day 45, there were 6 mice out of 16 (37.5%) from the HD group and 7 mice out of 16 (43.8%) from the LD group that achieved complete remission compared to 1 mouse out of 16 (6.3%) from the control group. Tumors in some mice in each of the groups developed ulcers that led to their removal from the study. Excluding the mice with tumor ulceration, we were able to analyze the overall survival of n = 13 mice in the isotype group and n = 12 mice in the LD group and n = 9 mice in the HD group (Fig. 4C). The median survival for the two treatment groups which was not reached at Day 45 is substantially higher than the isotope control group which was 26 days (Logrank P = 0.1199 for comparison with HD and 0.0957 for comparison with LD with a hazardous ratio [HR] of 2.836 and 2.459, respectively). Similar to tumor growth inhibition there was no significant difference in overall survival between the LD and HD SIWA318H treatment groups (Logrank P > 0.05, HR = 0.81). Overall, our results demonstrated the promising antitumor activity of SIWA318H as a single agent in a pancreatic cancer xenograft model in humanized NSG mice.

### SIWA318H depletes senescent cells and reduces fibrosis in tumor microenvironment

**Figure 5:**
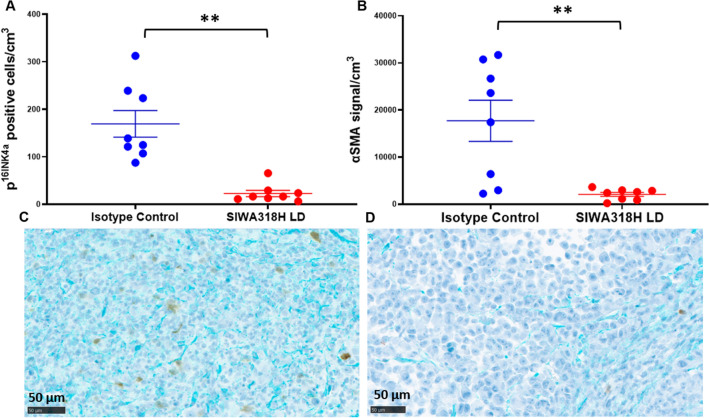
SIWA318H treatment depletes p^16INK4a^ positive cells and reduces αSMA expression in PSN1 xenograft tumors. (**A**) Quantification of the number of p^16INK4a^ positive cells in isotype control antibody treated and SIWA318H treated tumors. (**B**) Quantification of αSMA positive areas in isotype control antibody treated and SIWA318H treated tumors. (**C**) A representative image of tumor tissues from mice treated with isotype control antibody stained with antibodies against p^16INK4a^ (Brown) and αSMA (Teal). (**D**) A representative image of tumor tissues from mice treated with low dose SIWA318H stained with antibodies against p^16INK4a^ (Brown) and αSMA (Teal). Scale bar in (**C**) and (**D**) = 50 µm. **, p < 0.005.

To investigate the effects of SIWA318H treatment on PSN1 tumors we examined the expression of alpha smooth muscle actin (αSMA), a fibrosis marker, and p16^INK4a^, a cellular senescence marker, in tumor tissues harvested from mice treated with either isotype control antibody or LD SIWA318H using immunostaining. As shown in Fig. 5, compared to those treated with the isotype control antibody, SIWA318H treated tumors had significantly lower number of p16^INK4a^ positive senescent cells (Fig. 5A,C,D, p = 0.0002). In addition, the relative αSMA rich areas within the SIWA318H treated tumors were significantly smaller (p = 0.0031) compared to those of isotype control treated tumors, indicating that SIWA318H treatment significantly reduced tumor fibrosis (Fig. 5B–D).

## Discussion

Cells in a senescent state are dysfunctional cells that are arrested in their progress through the cell cycle, e.g., in the G1 Phase of the cell cycle, with an elevated level of p16^INK4a^ gene expression. All somatic cells that have the ability to divide can enter a state of senescence as a result of a plethora of stresses, including the stress of repeated cell division, genetic dysfunction, and oxidative stress (including oxidative stress due to aging or damage). Senescence is, except in the case of mutation-based senescence escape, a stable cell cycle arrest resulting from cellular damage, including aging-associated damage^14^. Although senescent cells do not divide, they are glycolytic and highly active metabolically^15^, e.g., they secrete a high level of inflammatory cytokines and growth factors comprising the so-called SASP which gives rise to an inflammatory pro-tumorigenic tumor microenvironment. The evidence of the effect of senescence on malignancy supports autonomous senescent cell suppression of malignancy^16^ and nonautonomous senescent cell promotion of malignancy^17^. Hence, in recent years targeting senescent cells as an approach for treating cancer and other aging related diseases has attracted significant attention^18^. Several strategies including senolytics, SASP inhibition, and immune elimination have been proposed and studied for eliminating senescent cells^19^.

One of the hallmarks of cellular senescence is the endogenous production of AGEs including CML that modify proteins. Hence, AGEs have been considered viable targets for targeting senescent cells^20,21^. The majority of AGE-targeted agents currently being developed are small molecules that either prevent the formation of AGEs, eliminate AGEs, or inhibit/modulate AGE response^22^. Although they have shown some promising activity in clinical trials for diabetic cardiovascular disease, these molecules were found to cause significant side effects and have yet to demonstrate meaningful clinical benefits in cancer^21,23^. There have been reports of antibodies that specifically recognize CML and/or CML glycated proteins^24–28^; however, they are mostly used for the detection and measurement of AGE accumulation in blood or tissues. Hence, SIWA318H reported here is, as far as we are aware, the first humanized CML glycation targeted antibody that shows promising activity in preclinical models for cancer.

SIWA318H was found to react with both cancer cells and cancer-associated fibroblasts in vitro and in vivo (Fig. 2) with mainly membranous and cytoplasmic, but occasional nuclear staining patterns. This finding is consistent with those reported for another CML-targeted monoclonal antibody that showed positive staining in cell membranes, cytoplasm, and nuclei of both cancer and non-cancer cells in pancreatic tumor tissues^26^. These results suggest that CML-targeted antibodies can potentially eliminate/inhibit both senescent cancer cells and stromal cells (e.g., CAFs) that support cancer cells. The removal of these cells, which reduces the production of SASP factors, can in turn inhibit the growth/function of other cancer cells and CAFs and lead to the suppression of tumor growth. The suppression of CAFs can modulate the tumor microenvironment and further improve therapeutic efficacy. The finding that SIWA318H reduced the expression of fibrosis marker αSMA in the PSN1 mouse xenograft tumors supports this mechanism of action (Fig. 5B and D).

SIWA318H was also found to interact with human FcγRIIIa and induce ADCC in vitro (Fig. 3). Hence, it is most likely that the elimination of p16^INK4a^ positive cells (Fig. 5A and C) by SIWA318H in the humanized NSG mouse model, at least in part, resulted from the NK cell-mediated ADCC activity. However, it is possible that binding of SIWA318H to CML^+^ cells can directly induce cell death (e.g., apoptosis). Further studies are needed to confirm this mechanism of action.

SIWA318H showed promising antitumor activity in the PSN1 xenograft model, particularly the impressive improvement in the complete response rate (Fig. 4), and supports its further development for the treatment of pancreatic cancer. Although the tumor growth inhibition was slightly greater in the high dose group compared to the low dose group, the difference was not statistically significant (Fig. 4). This could be due to either the dose for the low dose group being not low enough or the number of mice in each group being insufficiently powered to detect the small difference. In addition, the dense desmoplasia of pancreatic cancer has been shown to impede drug delivery and is considered a major challenge in pancreatic cancer treatment^29,30^. The significant reduction in αSMA expression in SIWA318H treated tumors suggests that SIWA31H can reduce pancreatic tumor fibrosis thus improving drug delivery and possibly immune infiltration. Furthermore, it has also been reported that chemotherapeutics can induce cellular senescence and drive treatment resistance and tumor progression^31^. Therefore, combination therapies that combine SIWA318H with chemotherapy and/or immunotherapy could exert even greater antitumor activity.

Finally, besides cancer, cellular senescence has been described in a number of diseases such as diabetes, lung and liver diseases, and neurodegenerative diseases, as well as some disease-induced conditions such as cachexia. In addition, a higher number of senescent cells, which are thought to play an important role in the aging process and the onset of chronical diseases, have been identified in tissues of older individuals^32,33^. Preclinical studies have demonstrated that eliminating senescent cells in aged mice exhibited a markedly improved health span^34,35^. Hence, we envision that cellular senescence targeted agents, particularly biologics like SIWA318H that have low toxicity, will have a broad clinical application beyond pancreatic cancer. We are currently exploring the utility of SIWA318H in some of these conditions.

### Supplementary Information

Supplementary Figures.

## Data Availability

All data generated or analyzed during this study are included in this published article and its Supplementary Information files.
