# Supplementary material for: Advanced glycation end product (AGE) targeting antibody SIWA318H is efficacious in preclinical models for pancreatic cancer
Source: Sci Rep. 2023 Oct 7;13:16953. doi: 10.1038/s41598-023-44211-6 (PMC10560265; doi:10.1038/s41598-023-44211-6)

**Supplementary Figure S1. Concentration dependent binding of SIWA318H and SIWA318M to CML-BSA.** SIWA318H and SIWA318M showed an almost identical concentration-dependent binding to CML-BSA. The binding assay was carried out using the same procedure as described for that of Figure 1. Each antibody concentration was assayed in triplicates.

**Supplementary Figure S2. Binding kinetics of SIWA318H to CML-BSA using surface plasmon resonance (SPR).** (A) SPR sensorgrams of SIWA318H binding to CM-BSA at 3 different concentrations (10, 100, and 500 nM). SIWA318H shows robust binding to CML-BSA at all 3 concentrations. (B) SPR sensorgrams for SIWA318H binding to BSA. SIWA318H shows no binding to BSA at any of the 3 concentrations tested. RU: Resonance Unit. The SPR assay was carried out using a Biacore T200 system.

Supplementary Figure S1

Supplementary Figure S2


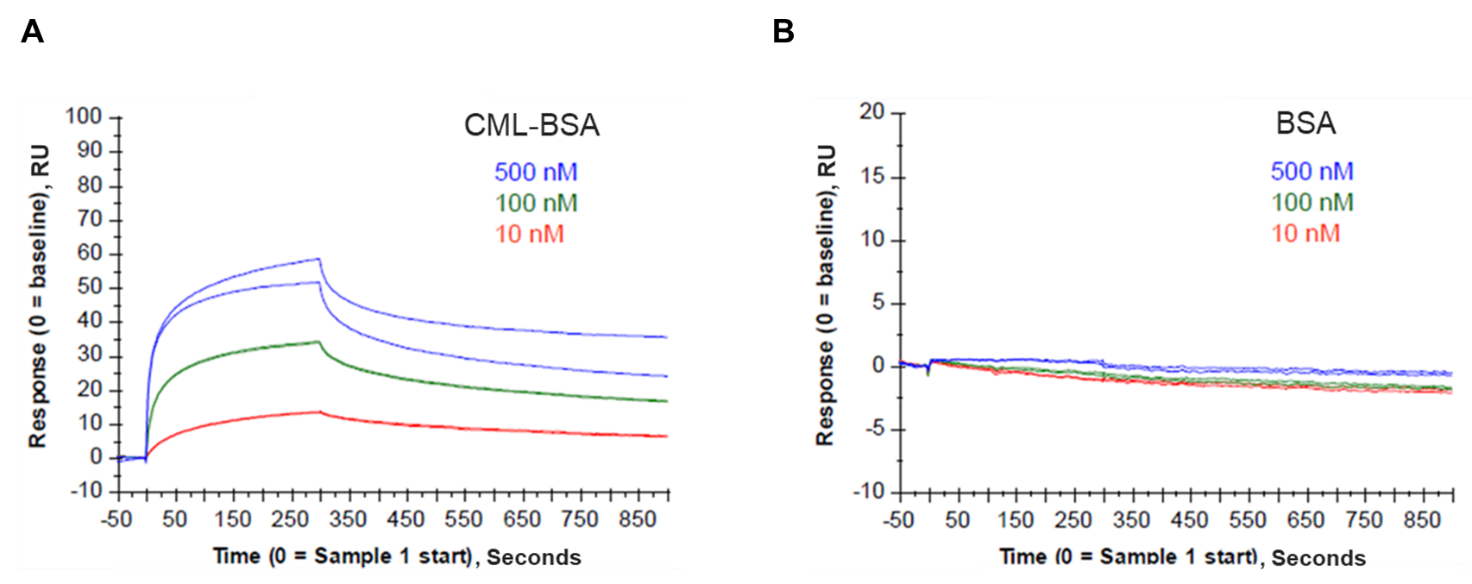

Supplement: Supplementary file 1 — Supplementary Figures. [file 41598_2023_44211_MOESM1_ESM.docx]
